# Microstructural Cortical Gray Matter Changes Preceding Accelerated Volume Changes in Individuals at Clinical High Risk for Psychosis

**DOI:** 10.21203/rs.3.rs-3179575/v1

**Published:** 2023-09-28

**Authors:** Ik Cho Kang, Ofer Pasternak, Fan Zhang, Nora Penzel, Johanna Seitz-Holland, Yingying Tang, Tianhong Zhang, Lihua Xu, Huijun Li, Matcheri Keshavan, Sue Whitfield-Gabrielli, Margaret Niznikiewicz, William Stone, Jijun Wang, Martha Shenton

**Affiliations:** Harvard Medical School; Harvard Medical School; Brigham and Women’s Hospital; Brigham and Women’s Hospital; Brigham and Women’s Hospital and Massachusetts General Hospital, Harvard Medical School; Shanghai Mental Health Center, Shanghai Jiao Tong University School of Medicine; Shanghai Mental Health Center, Shanghai Jiaotong University School of Medicine, Shanghai Key Laboratory of Psychotic Disorders, Shanghai 200030, PR China; Beth Israel Deaconess Medical Center; Northeastern University; Shanghai Key Laboratory of Psychotic Disorders, Shanghai Mental Health Center, Shanghai Jiao Tong University School of Medicine; Harvard Medical School

**Keywords:** gray matter microstructure, gray matter macrostructure, diffusion magnetic resonance imaging, ultra high risk

## Abstract

Recent studies show that accelerated cortical gray matter (GM) volume reduction seen in anatomical MRI can help distinguish between individuals at clinical high risk (CHR) for psychosis who will develop psychosis and those who will not. This reduction is thought to result from an accumulation of microstructural changes, such as decreased spine density and dendritic arborization. Detecting the microstructural sources of these changes before they accumulate is crucial, as volume reduction likely indicates an underlying neurodegenerative process. Our study aimed to detect these microstructural GM alterations using diffusion MRI (dMRI).

We tested for baseline and longitudinal group differences in anatomical and dMRI data from 160 individuals at CHR and 96 healthy controls (HC) acquired in a single imaging site. Eight cortical lobes were examined for GM volume and GM microstructure. A novel dMRI measure, interstitial free water (iFW), was used to quantify GM microstructure by eliminating cerebrospinal fluid contribution. Additionally, we assessed whether these measures differentiated the 33 individuals at CHR who developed psychosis (CHR-P) from the 127 individuals at CHR who did not (CHR-NP).

At baseline the CHR group had significantly higher iFW than HC in the prefrontal, temporal, parietal, and occipital lobes, while volume was reduced only in the temporal lobe. Neither iFW nor volume differentiated between the CHR-P and CHR-NP groups at baseline. However, in most brain areas, the CHR-P group demonstrated significantly accelerated iFW increase and volume reduction with time than the CHR-NP group.

Our results demonstrate that microstructural GM changes in individuals at CHR have a wider extent than volumetric changes and they predate the acceleration of brain changes that occur around psychosis onset. Microstructural GM changes are thus an early pathology at the prodromal stage of psychosis that may be useful for early detection and a better mechanistic understanding of psychosis development.

## Introduction

Schizophrenia, a severe psychotic disorder, typically emerges in late adolescence and early adulthood, leading to long-term disability [1]. To prevent the onset of psychosis, a concept of clinical-high risk (CHR) was introduced, which identifies individuals in a potential prodromal phase, allowing for careful monitoring and early intervention [2, 3]. Individuals at CHR exhibit attenuated positive symptoms and often experience ongoing neurocognitive and functional impairments which may obstruct their personal development during a crucial period [6, 7]. It has been observed that between 10–30% of individuals at CHR will develop a psychotic disorder within three years of their baseline assessment [3, 4].

With advances in technology, non-invasive brain imaging, particularly MRI, has emerged as a critical tool to study brain alterations in individuals at CHR. It is believed that early identification of these changes could provide important insights into the underlying mechanisms of psychosis. Consequently, several neuroimaging-derived measures have been proposed as potential early biomarkers for pathological changes and to monitor these changes over time [8, 9].

Anatomical MRI has been an important tool to detect macrostructural gray matter (GM) abnormalities, such as lower cortical volume, thickness, and gyrification [10–13]. Anatomical MRI studies of individuals at CHR report an accelerated decrease over time in cortical GM volume and thickness in those who later develop psychosis, compared to healthy controls (HCs) [16–19]. Importantly, similar but more extensive cortical abnormalities are found in later, more chronic stages of schizophrenia [14, 15]. This suggests that the macrostructural brain pathology is closely related to psychosis onset and may indicate atrophy-related degeneration processes that increase with the duration of the illness.

The underlying cause of these atrophy-related changes occurring around the onset of psychosis remains unclear, but it is thought to reflect accumulated alterations at the cellular level. Postmortem studies, for instance, have reported reduced somal sizes of pyramidal neurons [20–24], lower spine density, and less dendritic arborization [25–27] in schizophrenia, all of which could account for GM volume reductions [28–30]. It is hypothesized that such microstructural cellular changes might accumulate before the manifestation of detectable MR macrostructural changes, potentially serving as an earlier sign of an unfavorable clinical trajectory. Considering that atrophy-related changes likely indicate irreversible degeneration, identifying their microstructural sources before they further accumulate is crucial. Detecting these GM alterations in the CHR stage could enhance psychosis detection, deepen our understanding of its pathology, and potentially contribute to the development of new interventions.

Diffusion MRI (dMRI) is an in-vivo method that is sensitive to the micron-scale movement of water molecules within brain tissue, which makes it possible to detect subtle microstructural changes [31]. The majority of dMRI studies, however, have been focused on white matter (WM), for which current analysis tools are designed. These investigations have revealed WM abnormalities in individuals at CHR, irrespective of whether or not they eventually developed psychosis [32–35]. Moreover, a longitudinal WM study identified age-related WM abnormalities in CHR that appear to occur concurrently with development [36]. Early WM disruption also aligns with the finding of earlier WM maturation peak in individuals diagnosed with schizophrenia compared to HCs, in most brain regions [37]. Thus, unlike the macrostructural GM abnormalities, these microstructural WM alterations seem to be present even before psychosis onset and are less variable during psychosis progression. This could indicate an early WM microstructural deficit that might be a sign of predisposition to attenuated psychotic symptoms.

Among the measures derived from dMRI, free-water (FW) indicates the proportion of water molecules moving freely in the extracellular space of tissues. Prior studies have reported elevated FW levels around the time of psychosis onset, suggesting active pathological changes occurring around that period [38, 39]. Recent schizophrenia studies have extended FW analysis to the GM and found elevated FW that gradually decreases over time in the GM of individuals after their first psychotic episode [40]. These findings of FW in GM echo similar results in the WM, where FW elevation appears to peak around psychosis onset [41–43]. This temporal trajectory of FW changes implies active pathological processes that could predate volumetric changes in psychosis, such as neuroinflammation or other destructive processes that reduce microstructural barriers to water molecule diffusion, potentially predating volumetric changes in psychosis [39, 40, 44].

Investigating microstructural changes in cortical GM using dMRI has been a challenge due to the cerebrospinal fluid (CSF) surrounding the complex arrangement of gyri and sulci. The partial volume with CSF in many GM voxels affects diffusion measures, such as FW, making it difficult to discern if previous findings of increased FW in the GM reflect cellular changes or abnormal macrostructural processes identified by the anatomical MRI measures. To address this, we implemented a novel dMRI analysis that eliminates the CSF contribution from FW, resulting in an estimation of interstitial free-water (iFW).

Utilizing the iFW measure, this study aims to investigate whether microstructural GM abnormalities can be observed before macrostructural GM abnormalities in the CHR stage. We use longitudinal data from individuals at CHR and HCs, collected as part of the Shanghai-at-risk-for-psychosis (SHARP) program- the largest single-site dMRI study of the CHR stage to date. The dataset includes individuals at CHR who eventually developed psychosis (CHR-P) and those who did not (CHR-NP). We compared the iFW measure and anatomical MRI-derived volumetric measures across eight cortical lobes in the CHR-P CHR-NP and HC groups at baseline and over time. Our objective is to characterize the relationship between macro- and microstructural changes prior to psychosis onset and throughout its progression. We hypothesize that increases in the GM microstructural measure can be detected prior to lower volume in the CHR stage and prior to the expected rapid volumetric decline in the CHR-P group.

## Materials and methods

### Participants and Clinical Procedures

All data in this study were acquired at the Shanghai Mental Health Center (SMHC), Shanghai, China, as part of the SHARP program, which is NIMH-funded study directed by researchers in the United States and China (see Zhang et al. [45] for a detailed description of the program). Help-seeking individuals at CHR were recruited from clinic-wide questionnaire screening and clinician referrals. All individuals in the CHR group met the criteria for CHR at recruitment, defined by the Chinese version of the Structured Interview for Prodromal Syndromes (SIPS) and the Scale of Prodromal Symptoms (SOPS) [46–48], administered by a senior psychiatrist. The Global Assessment of Functioning Scale (GAF) [49] was administered at the time of recruitment and retrospectively for the previous twelve months (GAF-previous). Functional decline (per SIPS definition) was calculated as the drop in the current GAF score compared with the individual’s highest GAF score in the previous twelve months [50, 51].

The study had a longitudinal design with a baseline assessment and follow-up assessments three months following baseline and at yearly intervals for up to five years. Conversion to psychosis was evaluated or verified at every assessment using the SIPS “presence of psychotic symptoms” criteria [52]. The conversion date was estimated based on the information collected during the clinical interview. Accordingly, all individuals who eventually developed psychosis during the study were assigned to the CHR-P group, and the remaining individuals at CHR were assigned to the CHR-NP group. HC subjects were recruited through online advertisements. Exclusion criteria at study entry for all participants included head injury with loss of consciousness of any duration; any history of substance use, neurological disease, severe somatic diseases, IQ below 70, or dementia; and age younger than twelve or older than 35 years. HC subjects were additionally excluded if they met the criteria for a psychotic disorder, CHR syndrome (determined by the SIPS), or any other mental disorder defined by DSM-IV.[49] The study protocol and consent form were reviewed and approved by the local ethics committee at the SMHC and the Beth Israel Deaconess Medical Center. Written informed consent was obtained from all participants.

The sample had low illicit drug abuse prevalence and mostly homogeneous Han Chinese ethnicity, reflecting Shanghai’s population. Importantly, individuals at CHR were free from antipsychotics at enrollment, allowing for the investigation of brain changes directly associated with pathology rather than drug responses. Additionally, unlike many other large CHR studies, all data, including imaging, were collected at a single site, circumventing common technical challenges involving data harmonization across sites.

**Figure 1:**
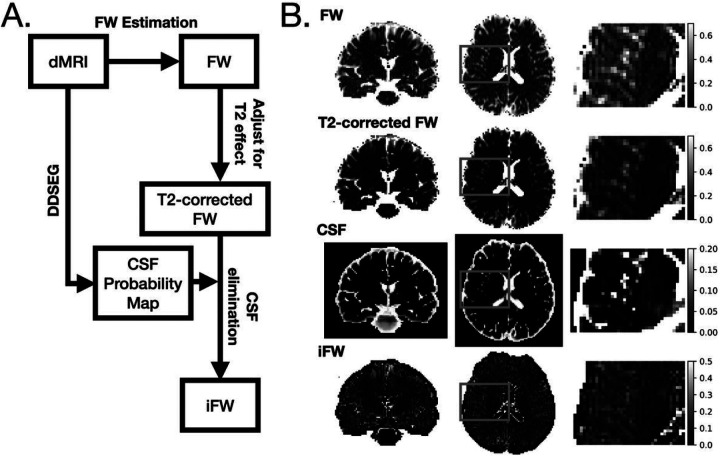
Estimation of iFW. (A) The steps involved in estimating iFW from dMRI data. (B) Example slice snapshots of FW, T2-corrected FW, CSF map from DDSeg, and iFW. FW, Freewater, iFW, interstitial Freewater.

For this study, we included all subjects who had T1-weighted (T1w) and dMRI available. After excluding low-quality data points due to motion artifacts or preprocessing issues, the final dataset included a longitudinal dataset of 160 individuals at CHR, with 33 in the CHR-P group, and 127 in the CHR-NP group (Table. 1). Additionally, there were 96 demographically matched HCs. The analyses included a total of 295 CHR scans and 175 HC scans. Refer to **ementary Fig. 1** for data exclusion details and **Supplementary Fig. 2** for the final scan count per assessment. The CHR-P group had an average of 395 ± 341 days from the first assessment to conversion (**Supplementary Fig. 3**). The age range at the baseline assessment was 13 to 34 years for the CHR group and 12 to 35 years for the HC group.

Although all individuals at CHR were free from antipsychotics at enrollment, 26 of the 160 individuals at CHR received antipsychotics between study enrollment and the baseline MRI scan. Five individuals at CHR were medicated over a month, while 21 individuals at CHR were medicated less than a month before their baseline MRI scan. (See **Supplementary Table 1**. for medication type and duration)

### MRI Acquisition and Processing

MR scans were acquired on a 3T MR system (Siemens, Verio) at SMHC with a 32-channel head coil. T1w images were acquired with the following parameters: a repetition time (TR) = 2300 ms, echo time (TE) = 2.96 ms, flip angle = 9 degrees, a field of view = 256 mm, and voxel size = 1 mm × 1 mm × 1 mm. For dMRI, parameters included TR = 15800 ms, TE = 109 ms, flip angle = 90 degrees, voxel size = 2 × 2 × 2 mm^3^ with b-values of 0, 200, 500, and 1000 s/mm^2^ with 5, 3, 6, and 30 volumes, respectively. An additional 30 gradient directions at b = 3000 s/mm^2^ were collected for tractography studies, but not used in FW calculations to avoid non-Gaussian effects.

### Image Processing

**Figure 2:**
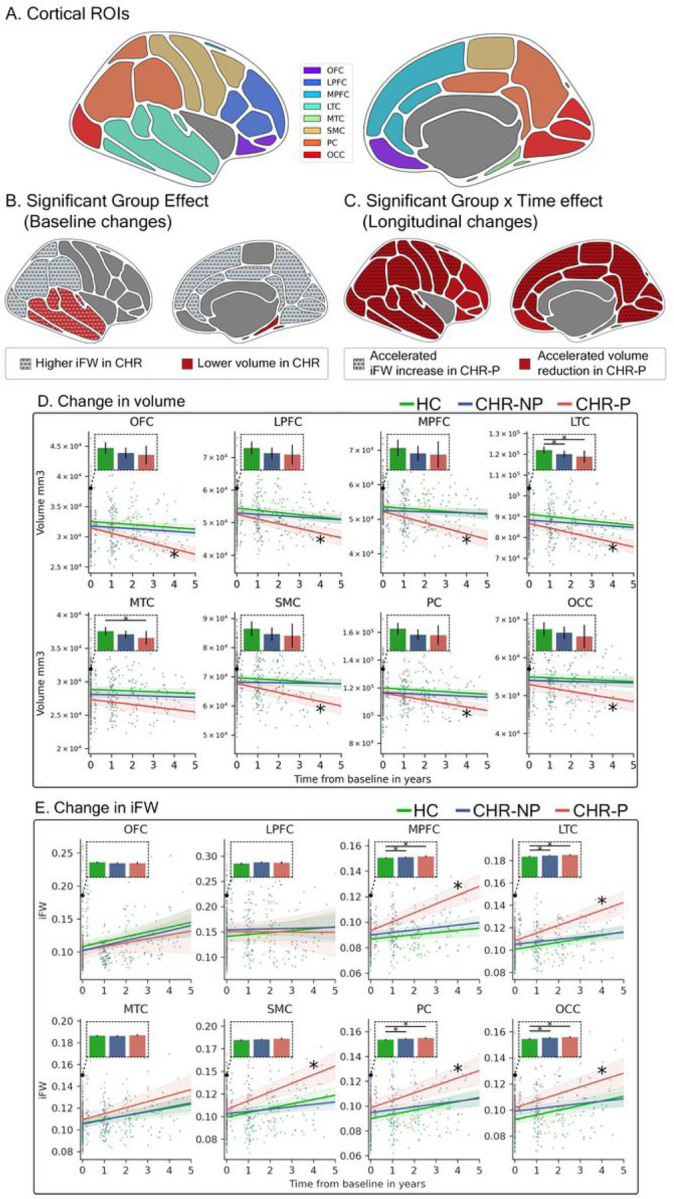
Baseline and longitudinal changes in iFW and volume. **(A)** Regions spanning eight cortical lobes were defined: OFC, orbitofrontal cortex, LPFC, lateral prefrontal cortex, MPFC, medial prefrontal cortex, LTC, lateral temporal cortex, MTC, medial temporal cortex, SMC, somatomotor cortex, PC, parietal cortex, OCC, occipital cortex. **(B)** At baseline, prior to psychosis onset, volume in CHR was significantly lower than in HC (red background) in the LTC and MTC. Significantly higher iFW in CHR compared to HC (dotted circles) were in MPFC, LTC, PC, and OCC, demonstrating a wider extent than the volume changes. **(C)**Longitudinally, increased rate of volume decline in CHR-P (red background) was found in all regions but the MTC. Increased rate of iFW change over time in CHR-P was found in MPFC, LTC, SMC, PC, and OCC (dotted circles). **(D** and **E)** The iFW and volume values, respectively, are plotted for each subject in each cortical region and time point. Lines represent the slopes estimated by each LME model and the shaded area around the lines represent 95 % confidence intervals. * marker on the slope line represents a significantly different rate of change in CHR-P compared to HC. Additional bar plots for each model present the intercepts which model baseline values, where the error bars represent 95% confidence intervals and the horizontal lines with * denote significant differences in the post hoc tests. Orbitofrontal, OFC, lateral prefrontal, LPFC, medial prefrontal, MPFC, lateral temporal, LTC, medial temporal, MTC, somatomotor, SMC, parietal, PC, occipital cortex, OCC, iFW, interstitial free-water, CHR, individuals at clinical high risk, HC, healthy controls.

FreeSurfer, version 6 [53, 54] with Desikan-Killiany cortical labels, was used to extract Intracranial volume (ICV) and to delineate eight cortical GM regions of interest (ROIs) from T1w (Fig. 2.A.): Orbitofrontal (OFC), lateral prefrontal (LPFC), medial prefrontal (MPFC), lateral temporal (LTC), medial temporal (MTC), somatomotor (SMC), parietal (PC), and occipital cortex (OCC). See **Supplementary Table 2** for the composition of each cortical region. ANTs [55] was used to register ROIs non-linearly from the T1 space to the diffusion space. FSL’s Eddy (version 6.0.1) with outlier replacement was used to correct head motion, eddy current, and any outlier slices using Gaussian Process modeling [56]. FW maps were estimated using in-house Matlab script [57].

### Interstitial free-water

The FW measure represents the fractional volume of extracellular water, which is composed of water molecules in the interstitial FW, but also other extracellular water molecules such as plasma, and CSF [58]. Deep dMRI Tissue Segmentation (DDSeg) [59], a machine learning-based tissue segmentation method, was used to estimate the fractional volume of CSF (CSFv) in each voxel directly in the diffusion space. An Interstitial FW (iFW) measure was calculated by assuming a negligible contribution of plasma, estimating the CSFv, and subtracting this amount from the FW value, i.e., iFW = FW – CSFv. Negative iFW values were projected to zero. Compensating FW for CSFv was first suggested in Montal et al. [60]. Here, to improve comparability and reduce bias we modified the estimation of CSFv and FW. More specifically, CSFv was calculated from DDSeg as described above, and the FW value was calculated by compensating the FW maps for T2 relaxation differences between CSF and GM (see **Supplementary Methods 1**), reducing overestimation bias due to the T2 weighting expected in the two-compartment model fit [57]. See Fig. 1 .A. for a schematic diagram describing the process of deriving iFW from dMRI. Subtracting the CSF contribution mainly affects voxels near the ventricles or at the interface of GM and CSF (Fig. 1 .B.). iFW was averaged for each cortical ROI in the diffusion space for each subject.

### Statistical analyses

For statistical tests we used Statsmodels [61] in Python if not stated otherwise.

### Group comparisons

ANOVA was used to test for group effects in age, education, GAF, and motion. Chi-square test was used to compare the difference in the sex ratios between groups, and T-test was used to compare baseline SIPS between CHR-NP and CHR-P To compare the volume and iFW of each cortical ROI, a linear mixed effects (LME) model was used with a random subject effect and fixed effects for group, time from baseline in years, and group and time interaction. Age, sex, and intracranial volume were included as covariates. The model was implemented using the lme4 package [62] in R (4.0.5) [63] with the following formula: “measure ~ Group+ Time-from-baseline + (Group * Time-from-baseline) + age + sex + ICV”, and a random intercept effect grouped by Subject. The “Group” variable included three categories, HC, CHR-P and CHR-NP and its effect represents differences in the measure at baseline (i.e., at Time-from-baseline = 0). The interaction effect represents longitudinal group differences (i.e., group differences in the rate of change of a measure). Tukey’s post hoc test in R’s Emmeans package was used to test for group differences between any two groups in ROIs with significant group effects. ANOVA tests were corrected for multiple comparisons using the false discovery rate (FDR) correction for eight cortical ROIs.

Since some subjects included time points that were two years or more following baseline, we performed additional analyses to investigate the possible influence of time-from-baseline (see **Supplementary Analysis 1**).

### Correlation of imaging measures with clinical variables

We used Spearman correlation to identify clinical associations of iFW and volume at baseline across the whole CHR group. The correlation tests included the sum of all SIPS scores (SIPS total), the sum of each sub-category of SIPS (SIPS-P Positive; SIPS-N, negative; SIPS-D, disorganized; and SIPS-G, general symptoms), and the GAF score. These tests were applied in ROIs with the significant Group effects in preceding analyses. Similarly, the rate of change in iFW or volume was also tested for correlations against the rate of change in clinical variables across the CHR-P group using Spearman correlation in those ROIs with significant Group * Time interaction. This analysis included 17 CHR-P individuals who had at least two MRI scans. All Spearman correlation tests were corrected for multiple comparisons using FDR correction.

### Prediction of conversion to psychosis from baseline imaging data

As a proof-of-concept analysis, we trained support vector machines [64] using the baseline data to predict which individual at CHR will develop psychosis. We compared prediction models with input of iFW only, volume only, and iFW and volume combined (see **Supplementary Analysis 2** for details).

### Correlation between macro- and microstructural imaging measures

We tested for associations between the rates of changes in iFW and cortical volume using Spearman correlation. Using Fisher’s z method implemented in the Cocor package, we also compared the correlation between the rates of change in iFW and cortical volume in CHR-P compared to CHR-NP The rate of change in volume and iFW, in each cortical ROI, was estimated as the slope of linear fit of each individual’s longitudinal data. This analysis included subjects with at least two MRI scans (45 HC; 66 CHR-NP; 17 CHR-P). We used FDR correction for the multiple comparisons across the eight cortical ROIs. Please see Supplementary Figs. 6 and 7 for CSF elimination effect evaluation.

## Results

### Demographic and clinical information

There were no significant group differences in age, sex, or education between the CHR-NP CHR-P and HC groups at baseline (see Table 1 for details). There were no significant differences in the baseline SIPS scores between the CHR-NP group and the CHR-P group, although, as expected, SIPS scores decreased over time for the CHR-NP group, and increased for the CHR-P group (**Supplementary Fig. 4**).

At baseline, ANOVA indicated significant group differences in current GAF and the highest GAF in the previous 12 months. Post hoc tests showed higher scores in the HC group and no significant differences in GAF measures between the CHR-NP and CHR-P groups. There were also no significant group differences in dMRI absolute or relative motion.

### Macrostructural GM group comparison: cortical volume

The LME model for the volume measure showed a significant Group effect, indicating baseline volume differences in two of the eight cortical ROIs (Fig. 2.B.): LTC and MTC. Post hoc Tukey tests showed that the CHR-P group had significantly lower volumes in both ROIs compared to the HC group, while the CHR-NP group showed a significantly lower volume only in the LTC compared to the HC group. There were no significant volume differences between the CHR-P and CHR-NP groups at baseline (Table 2).

The LME model also identified significant longitudinal group differences in all ROIs, except the MTC (Fig. 2.C.). Post hoc Tukey tests indicated an accelerated cortical volume decrease over time in the CHR-P group compared to both HC and CHR-NP groups (Fig. 2.D.).

### Microstructural GM group comparison: iFW

The LME model for the iFW measure showed a significant Group effect, indicating baseline iFW differences, in four of eight cortical ROIs (Fig. 2.B.): MPFC, LTC, PC, and OCC. Post hoc Tukey tests showed that both CHR-NP and CHR-P groups had greater baseline iFW compared to the HC group in all four ROIs. There were no significant iFW differences between the CHR-P and CHR-NP groups at baseline (Table 3).

The LME model also identified significant longitudinal iFW group differences in five ROIs: MPFC, LTC, SMC, PC, and OCC. (Fig. 2.C.) Post hoc tests in these ROIs revealed that the CHR-P group showed a significantly accelerated iFW increase over time compared to the CHR-NP group. For the MPFC, LTC, and SMC ROIs, the rate of iFW increase over time in the CHR-P group was also higher than that of the HC group. (Fig. 2.E.).

**Supplementary Analysis 1** further demonstrated that the longitudinal effects were not driven by those subjects who had assessments completed more than two years following baseline.

### Correlation of imaging measures with clinical variables

To establish clinical implications of the abnormalities found in the volume and iFW measures, we further investigated correlations with clinical scores across the entire CHR group (i.e., the CHR-P and CHR-NP groups combined) in only those brain regions that showed significant volume (LTC and MTC) or iFW (MPFC, PC, OCC, and LTC) baseline differences.

### Correlations at baseline

**Figure 3:**
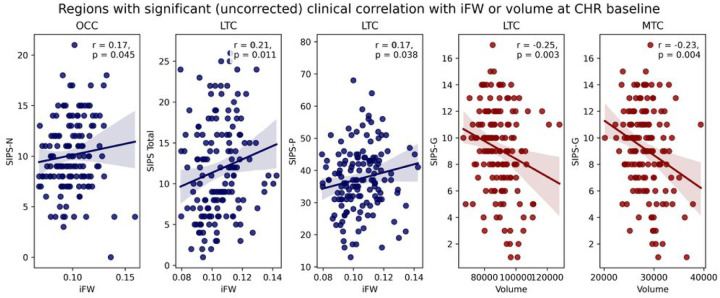
Correlations with clinical scores at the baseline assessment. Scatter plots present the relationship at the baseline assessment between iFW (blue) or volume (red), and clinical variables across CHR-NP and CHR-P grouped together. Solid lines represent the Spearman correlation coefficient. SIPS, Structured Interview for Prodromal Syndromes, SIPS-N, SIPS negative symptom scores, SIPS-P SIPS positive symptom scores, SIPS-G, SIPS general symptom scores. The p-values are not corrected for multiple comparisons.

None of the correlations between baseline imaging measure and baseline clinical scores were significant after multiple comparison corrections. Several correlations were significant before multiple comparison corrections (Fig. 3): LTC volume (*r*=−.25, *P*= .003) and MTC volume (*r*=−.23, *P*= .004) with SIPS-G, OCC iFW with SIPS-N (*r*= .17, *P*= .045), LTC iFW with SIPS-P (*r*= .17, *P*= .038) and with SIPS total scores (*r* = .21, *P*=.011).

### Correlations between longitudinal rates of change

None of the correlations between the rate of change in iFW or volume, and the rate of change of clinical variables across the CHR-P group were significant after multiple comparison corrections. For completeness, several correlations that were significant before multiple comparison corrections are reported in **Supplementary Fig. 5**.

### Prediction of conversion to psychosis from baseline imaging data

The model that included iFW alone outperformed the model that included volume alone, or the model that included both iFW and volume. The prediction power of all models was, however, low; the prediction using iFW was nonetheless statistically better than chance (balanced accuracy: 59.8%, area under the curve: 0.56, *P*= .032; see **Supplementary Analysis 2** for details).

### Correlation between iFW and volume

At baseline, there was no significant correlation between the volume and iFW in any cortical ROI in any group (**Supplementary Fig. 5**). For the longitudinal rate of changes (**Supplementary Fig. 8**), the HC group showed significant negative correlation between the rate of change in volume and rate of change in iFW in the OCC (*r*=−.41, P_FDR_ = .035). The CHR-NP group showed significant correlations in the LTC (*r*= − .60, *P*_*FDR*_ < .001), and the CHR-P group showed significant correlations in the OFC (*r*= −65, *P*_*FDR*_ = −035) and OCC (*r*= − .82, *P*_*FDR*_ < .001) (**Supplementary Fig. 9**). In the OFC and OCC, the correlations were significantly stronger in the CHR-P group than in the CHR-NP or the HC groups (**Supplementary Fig. 10**). For the effect of CSF elimination on correlations, see **Supplementary Figs. 6 and 7**.

## Discussion

We found that microstructural GM abnormalities in the form of iFW exist before psychosis onset, and are more widespread than volumetric changes in individuals at CHR. Over time, and in overlapping regions, the CHR-P group shows accelerated volume reduction and iFW increase compared to the CHR-NP and HC groups. These results suggest that microstructural GM changes may characterize earlier brain alterations related to the emergence of symptoms, and before a more widespread brain volume reduction occurs around the onset of psychosis.

### Cortical Volume Changes in Individuals at CHR

Most previous GM studies of individuals at CHR focused on macrostructural changes, and demonstrated an accelerated volume reduction over time in the CHR-P group [17–19]. Similarly, in our data, the CHR-P group showed a significant accelerated decrease in volume over time in most cortical ROIs. It is thus clear that a marked reduction in GM volume occurs around psychosis onset. However, the longitudinal changes in the current study cannot inform whether the volumetric changes begin before or after onset, because most follow-up data points for individuals in the CHR-P group occur after psychosis onset (**Supplementary Fig. 2**). Therefore, assessing baseline findings is crucial for establishing the timing of brain volumetric reductions. Previous reports of volumetric changes in individuals at CHR before the conversion to psychosis are, however, conflicting, with some studies reporting lower volume [11, 12], while others reporting no changes [17, 18, 65, 66]. In our sample, we identified lower baseline GM volume in the CHR group that was limited to the temporal lobe (LTC and MTC) and lower volumes in LTC and MTC were correlated with higher SIPS scores (although below multiple comparison correction threshold), suggesting that volumetric changes in the temporal lobe may play an early role in the development of psychosis risk. Nevertheless, the lower baseline volume did not dissociate the CHR-P from the CHR-NP In addition, the lower volume at baseline was identified in a much smaller region than the area that showed accelerated volume reduction over time in the CHR-P group (found in all ROIs except the MTC). Taken together, these volumetric findings suggest that early volumetric changes may occur in the temporal lobe, while other areas of the brain may only show accelerated volume decrease closer to, and possibly after, psychosis onset.

### Microstructural Changes in CHR

Unlike baseline volumetric changes in the CHR group, which were limited to the temporal lobe, we find that iFW microstructural changes in the CHR group at baseline were more widespread, with higher iFW than the HC at the MPFC, LTC, PC, and OCC. The overlapping yet more extensive higher iFW than lower volume at baseline suggests that detectable GM microstructural changes precede detectable macrostructural GM changes at the CHR stage. The higher iFW at baselines was followed by accelerated iFW increases in the CHR-P group found in MPFC, LTC, PC, OCC, and SMC, which were also ROIs that showed accelerated volume reductions. Microstructural iFW changes at baseline in the CHR stage may thus represent early abnormalities in regions that later show accelerated changes around the time of psychosis onset. Higher iFW findings at baseline align with previous studies that report increased FW in the GM of individuals following their first-episode of psychosis [38–40]. See our **Supplementary Discussion** for insights on how microstructural changes in CHR patients’ specific brain regions precede macrostructural changes, suggesting early symptom development and the need for advanced MRI techniques.

### CHR-P vs CHR-NP

At baseline, both CHR groups were different from HC but the volume reduction and iFW increase did not distinguish between the CHR-P and CHR-NP groups. Therefore, we cannot yet determine that the microstructural abnormality in the CHR group predisposes individuals to develop psychosis. However, as shown in **Supplementary Analysis 2**, baseline iFW seems to have some utility in distinguishing between individuals from the CHR-P group and individuals from the CHR-NP group, where a prediction based on iFW performs better than chance level, and also better than a prediction based on volumetric measures. This is suggestive of a potential benefit in the inclusion of iFW in more elaborated models based on additional variables, aiming at the prediction of psychosis onset.

### Biological substrate for the increased iFW

The exact biological substrate for the iFW change in GM is complex to pinpoint. By eliminating CSF contribution, the iFW measure in GM voxels is expected to reflect other extracellular spaces, which include interstitial fluid, and plasma [57, 67, 68]. An increase in interstitial fluid in CHR aligns with previous postmortem findings of reduced cellularity in schizophrenia, such as smaller somal size, lower spine density, and dendritic arborization (see Moyer et al., 2015 for review) [69], which are also thought to lead to volumetric reductions in schizophrenia eventually [29, 30]. Such a reduction in the cellular domain is likely accompanied by a larger ratio of extracellular spaces in GM, which could explain an increased iFW. However, asserting a relationship between iFW and a reduction in the cellular domain requires post-mortem histological studies or appropriate animal models.

Previous studies in schizophrenia and psychosis suggested a potential link between increased FW and neuroinflammation [39, 42, 70, 71]. Neuroinflammation is implicated in schizophrenia [28, 72, 73], and it often leads to vasogenic edema, increasing water in the extracellular interstitial spaces. Our previous research found increased FW in the WM of maternal immune exposed rats, indicating inflammation [70] and a correlation between IL-6 and TNF-alpha with FW in WM in patients with schizophrenia [71]. Another study showed a negative correlation between glutathione, a major antioxidant in the brain, and FW in GM in schizophrenia [39]. Similarly, the increase in iFW in GM might reflect higher water content due to neuroinflammatory responses and should be examined alongside inflammatory markers in future studies. It would be valuable to investigate if iFW levels in GM decrease during chronic stages when the inflammatory response presumably subsides, similar to FW in WM [42].

### Technical considerations in iFW estimation

The iFW measure is designed to disentangle microstructural changes in interstitial water from changes that stem from macrostructural volume reduction. The ability to do so is demonstrated 1) by the lower absolute values of iFW relative to FW, 2) by the lack of significant correlations between iFW and volume at baseline, and 3) by the generally weaker correlation of iFW and volume compared to FW and volume. We note, however, that some biological relationship between micro- and macrostructural changes may still be expected (i.e., a biological process that changes both), which may explain the stronger correlation between the rate of change in iFW increase and volume reduction in the CHR-P group over time compared with that in the CHR-NP group.

The iFW measure requires an estimation of the CSF fraction and FW in the same voxel. We used DDSeg [59] to estimate the CSF fraction directly in the dMRI space, which provides more accurate segmentation than T1w-image tissue segmentation by circumventing co-registration with the T1w space [59]. However, we note that we used registration to transfer the cortical ROIs delineated on the T1w data into the diffusion space. As a result, inaccurate registration may still cause some GM ROIs to “spill” into neighboring non-GM voxels, potentially biasing their average iFW measures. Future studies may consider more advanced segmentation approaches to identify brain regions directly in the diffusion space.

We also applied a correction accounting for the expected T2 values of GM that are different from those of CSF, which reduces the overestimation of FW often seen in GM voxels. However, our model assumes a negligible contribution of plasma to the FW estimation, and a fixed value of GM T2. Future implementations may benefit from more accurate FW estimations by methods that simultaneously estimate the voxel-wise relaxation rates and diffusivities [74–76], which requires more elaborated dMRI data such as multi-dimensional acquisition [77], and/or models estimating the pseudo-diffusion effects of plasma from extremely low b-values [78].

### Longitudinal data limitations

Limitations of our study include the relatively small number of longitudinal follow-up data. Another limitation is the inclusion of data points acquired more than two years after the conversion to psychosis. These points may or may not reflect the same biological processes affecting the imaging data soon after the development of psychosis, and, therefore, may have altered some of the results. However, by reanalyzing the data without these points (in **Supplementary Analysis 1**) we showed that they had little effect on the results.

Another limitation of our data is the possible cumulative effect of medication on follow-up assessments. There is still much debate regarding the impact of antipsychotics on brain structure, with conflicting reports [79–83]. Our study participants were primarily unmedicated at first assessment, but over time, most of the individuals at CHR were treated with antipsychotics. The current study design does not allow the separation of longitudinal effects caused by medication response from those caused by pathological changes. This aspect remains to be investigated in future studies.

## Conclusion

In conclusion, we identified early GM microstructural changes in CHR, which were more widespread than volumetric changes, suggesting that detectable microstructural GM changes predate macrostructural changes in the prodromal stage of psychosis. In those who develop psychosis, we then found accelerated microstructural and macrostructural changes, suggesting a progressive pathophysiological change around psychosis onset. Our results thus emphasize the utility and importance of GM microstructure as an additional biomarker important for a better mechanistic understanding of the development of psychosis.

## Figures and Tables

**Table 1:** Demographic and clinical information at baseline

Value At baseline		CHR-NP (n = 127)	CHR-P (n = 33)	HC(n = 96)	Statistic	P
**Age (years)**		18.9 ±5.0	19.3 ± 5.1	18.6 ± 4.5	F = 0.24	0.79
**Education (years)**		10.7 ±3.0	10.1 ± 1.9	10.9 ± 2.3	F = 1.02	0.36
**Sex (M/F)**		59 / 68	22/11	51 / 45	X2 = 4.43	0.11
**Scale of Prodromal symptoms**	**Positive**	10.2 ± 3.5	10.3 ± 3.5		T = −0.2	−0.84
	**Negative**	11.5 ±6.1	12.5 ± 6.1		T = −0.77	0.44
	**Disorganization**	6.6 ±3.2	6.6 ± 3.3		T = 0.08	0.93
	**General**	9.3 ±3.2	8.7 ± 3.2		0.95	0.34
**Maximum Global Assessment of Functioning Scale in the past 12 months**		77.2 ±4.9	77.4 ± 2.9	80.8 ± 1.9	F = 25.3	**<0.0001**
**Global Assessment of Functioning Scale - current**		53.6 ±8.2	52.8 ± 7.3	80.4 ± 2.2	F = 517.9	**<0.0001**
**Number of subjects with medication more than a month**		3 subjects	2 subjects	-		
**Absolute motion in dMRI**		0.66 ± 0.29	0.63 ± 0.22	0.64 ± 0.32	F = 0.42	0.66
**Relative motion in dMRI**		0.30 ± 0.14	0.29 ± 0.11	0.30 ± 0.13	F = 0.22	0.80

**Table 2.** LME models- Volume. Group and Group X Time-from-baseline interaction effect on volume

Group effect on volume (Baseline difference between groups)					Post hoc (*t* and *P*)					
	DoF	F	P	P-FDR	CHR-P vs HC		CHR-NP vs HC			CHR-P vs CHR-NP
**OFC**	2,273	2.30	0.1022	0.2044						
**LPFC**	2,263	1.96	0.1425	0.2280						
**MPFC**	2,270	0.83	0.4378	0.4378						
**LTC**	2,264	6.74	0.0014	**0.0112**	3.33	**0.0028**	2.71	**0.0196**	1.56	0.2636
**MTC**	2,263	5.43	0.0049	**0.0195**	3.19	**0.0045**	1.99	0.1170	1.92	0.1351
**SMC**	2,263	1.47	0.2322	0.2973						
**PC**	2,263	3.38	0.0354	0.0945						
**OCC**	2,260	1.35	0.2601	0.2973						
Group x Time-from-baseline interaction effect on volume					Post hoc (*t* and *P*)					
	DoF	F	P	P-FDR	CHR-P vs HC			CHR-NP vs HC		CHR-P vs CHR-NP
**OFC**	2,239	11.47	0.0000	**0.0001**	4.52	**<0.0001**	0.37	0.9255	4.51	**< 0.0001**
**LPFC**	2,229	6.28	0.0022	**0.0030**	2.84	**0.0137**	−0.80	0.7059	3.54	**0.0014**
**MPFC**	2,237	12.92	0.0000	**0.0000**	4.21	**0.0001**	−0.90	0.6390	5.06	**< 0.0001**
**LTC**	2,229	8.88	0.0002	**0.0004**	3.47	**0.0018**	−0.78	0.7171	4.20	**0.0001**
**MTC**	2,226	2.62	0.0754	0.0754						
**SMC**	2,227	10.99	0.0000	**0.0001**	3.71	**0.0008**	−1.13	0.4995	4.68	**< 0.0001**
**PC**	2,228	7.05	0.0011	**0.0017**	3.36	**0.0027**	−0.19	0.9813	3.66	**0.0009**
**OCC**	2,223	4.34	0.0142	**0.0162**	2.65	**0.0236**	−0.12	0.9923	2.87	**0.0125**

**Table 3.** LME models- iFW. Group and Group X Time-from-baseline interaction effect on iFW

	Group effect on iFW				Post hoc (*t* and *P*)					
	DoF	F	P	P-FDR	CHR-P vs HC		CHR-NP vs HC		CHR-P vs CHR- NP	
**OFC**	2,344	0.01	0.9932	0.9932						
**LPFC**	2,361	0.40	0.6694	0.7650						
**MPFC**	2,288	4.99	0.0074	**0.0148**	−2.86	**0.0129**	−2.36	**0.0499**	−1.31	0.3889
**LTC**	2,295	5.42	0.0049	**0.0131**	−2.93	**0.0103**	−2.52	**0.0327**	−1.28	0.4082
**MTC**	2,327	1.97	0.1412	0.1882						
**SMC**	2,284	2.91	0.0560	0.0896						
**PC**	2,278	5.59	0.0042	**0.0131**	−2.94	**0.0098**	−2.60	**0.0264**	−1.24	0.4319
**OCC**	2,290	5.61	0.0041	**0.0131**	−2.76	**0.0171**	−2.82	**0.0140**	−0.89	0.6473
	Group x Time interaction effect on iFW				Post hoc (*t* and *P*)					
	DoF	F	P	P-FDR	CHR-P vs HC		CHR-NP vs HC		CHR-P vs CHR- NP	
**OFC**	2,389	0.09	0.9185	0.9185						
**LPFC**	2,377	0.23	0.7978	0.9118						
**MPFC**	2,262	12.71	0.0000	**<0.0001**	−4.63	**<0.0001**	−0.06	0.9982	−4.85	**<0.0001**
**LTC**	2,272	8.00	0.0004	**0.0011**	−3.01	**0.0081**	1.19	0.4610	−3.99	**0.0003**
**MTC**	2,321	1.06	0.3479	0.4639						
**SMC**	2,257	9.23	0.0001	**0.0005**	−2.59	**0.0272**	2.08	0.0955	−4.16	**0.0001**
**PC**	2,248	5.23	0.0060	**0.0119**	−2.00	0.1150	1.52	0.2847	−3.15	**0.0052**
**OCC**	2,265	3.64	0.0277	**0.0443**	−1.24	0.4310	1.69	0.2124	−2.47	**0.0380**
